# Identification of key regulators responsible for dysregulated networks in osteoarthritis by large-scale expression analysis

**DOI:** 10.1186/s13018-021-02402-9

**Published:** 2021-04-14

**Authors:** Song Shi, Fuyin Wan, Zhenyu Zhou, Ran Tao, Yue Lu, Ming Zhou, Fan Liu, Yake Liu

**Affiliations:** Department of Orthopaedics, Affiliated Hospital of Nantong University, Nantong, Jiangsu China

**Keywords:** Osteoarthritis, Transcriptional profiling, Drug repurposing

## Abstract

**Background:**

Osteoarthritis (OA) is a worldwide musculoskeletal disorder. However, disease-modifying therapies for OA are not available. Here, we aimed to characterize the molecular signatures of OA and to identify novel therapeutic targets and strategies to improve the treatment of OA.

**Methods:**

We collected genome-wide transcriptome data performed on 132 OA and 74 normal human cartilage or synovium tissues from 7 independent datasets. Differential gene expression analysis and functional enrichment were performed to identify genes and pathways that were dysregulated in OA. The computational drug repurposing method was used to uncover drugs that could be repurposed to treat OA.

**Results:**

We identified several pathways associated with the development of OA, such as extracellular matrix organization, inflammation, bone development, and ossification. By protein-protein interaction (PPI) network analysis, we prioritized several hub genes, such as *JUN*, *CDKN1A*, *VEGFA*, and *FOXO3.* Moreover, we repurposed several FDA-approved drugs, such as cardiac glycosides, that could be used in the treatment of OA.

**Conclusions:**

We proposed that the hub genes we identified would play a role in cartilage homeostasis and could be important diagnostic and therapeutic targets. Drugs such as cardiac glycosides provided new possibilities for the treatment of OA.

**Supplementary Information:**

The online version contains supplementary material available at 10.1186/s13018-021-02402-9.

## Background

Osteoarthritis (OA) is the most prevalent musculoskeletal disease and a leading cause of disability in the elderly [1, 2]. OA is characterized by cartilage degeneration, the formation of osteophytes, subchondral bone remodeling, and pathological changes of the meniscus and synovitis [3]. It causes pain, joint stiffness, and disability and leads to severe economic and social burden [4]. Pain is the dominant symptom and a significant driver of clinical decision-making [5]. Treatment of OA includes alleviating pain, controlling inflammation, and slowing down tissue degradation [6]. However, there is currently no effective pharmaceutical treatment for OA that can decelerate the progression of the disease since the precise mechanisms of the pathogenesis of OA remains largely undetermined [7].

The integration of genome-scale transcriptomic profiling of different patient cohorts improves the understanding of molecular changes during OA progression and provides a scientific rationale for the development of novel treatment strategies. RNA-seq and microarray technology are widely used high-throughput genotyping methods that measure the expression of genes on a genome-wide scale with high accuracy and reliability [8–10]. Previously, it has been reported that during the pathogenesis of OA, the molecular signature of articular cartilage and synovial membrane underwent huge changes [11]. Expression of genes involved in the molecular matrix components, cell–matrix adhesion, and ossification had dramatic changes, which typically marked different stages of endochondral ossification or transient cartilage differentiation [11–13]. These changes result in remodeling of the matrix and lead to the impaired function of the tissue [14]. Notably, local low-grade inflammation of articular cartilage and synovial from patients with OA was also observed. During the progression of OA, pro-inflammatory cytokines such as IL-1, IL-6, TNF or interferon [15], were secreted from diseased tissues, which further damaged surrounding tissues and led to cartilage degradation [7]. Therapeutic strategies aimed at countering inflammation is promising but does not arrest the progressive degeneration of articular cartilage [14, 16, 17]. Thus, identifying the molecular basis of OA progression and the development of novel therapeutic strategies to improve the treatment of OA remain the key issues in OA research.

To characterize OA at the molecular level and to uncover the pathogenesis mechanisms, we collected and integrated transcriptome data of hundreds of OA and normal tissues from 7 independent studies. By comparing the gene expression pattern of OA samples with normal samples, we identified significantly differentially expressed genes (DEGs) and investigated the enriched signaling pathways of these DEGs. Using protein–protein interaction (PPI) network analysis, we discovered several hub genes that function as critical regulators in OA development and might be ideal drug targets. Furthermore, we used a computational drug repurposing method to uncover several drugs that could be repurposed to treat OA.

## Methods

### Literature search and data collection

We used the keywords “Osteoarthritis”, “RNA-seq or microarray”, and “Dataset” on PubMed (https://www.ncbi.nlm.nih.gov/pubmed/), GEO (http://www.ncbi.nlm.nih.gov/geo/), or ArrayExpress (https://www.ebi.ac.uk/arrayexpress/) to find relevant publications or transcriptome datasets of OA. Transcriptome data of synovial or cartilage from OA patients were included in the analysis. In the data filtering and quality control process, we selected datasets with more than three replications for each condition and measuring over 10,000 genes to ensure the coverage of genes. Altogether, we collected 132 OA samples and 74 normal samples from 7 independent datasets for further analysis.

### Data normalization and removal of batch effects

For the microarray data, raw “.CEL” file was downloaded. R program (version 3.5.1; http://www.r-project.org) was used for data analysis. “oligo” [18] and “affy” [19] R packages were applied for the data processing, and then the Robust Multi-array Average (RMA) method [20] was used for background correction, normalization, and probe set summarization. For RNA-Seq data, the sequence data were aligned to the human reference genome hg38 using STAR (v2.5.3a). RSEM (v1.2.28) was applied to map aligned reads and to generate a gene count matrix by default parameters. The expression matrix was normalized using the quantile normalization method. Residual technical batch effects arising due to heterogeneous data platforms were corrected using the ComBat [21] function.

### Filtering of differentially expressed genes

For microarray data, “limma” [22] was used to perform the differential gene expression analysis of each dataset. For RNA-seq data, differential expression analysis was performed using Deseq2 [23]. False discovery rate (FDR) was applied to carry out the correction of multiple testing using the Benjamini and Hochberg (BH) method. In this study, genes with |log2fold change (FC)| > 1.2 and FDR < 0.1 were selected as the threshold for differentially expressed genes (DEGs). Genes that were differentially expressed in over half of the datasets with the sample condition were selected and used for further analysis. Gene set enrichment analysis for DEGs was performed using “MAGeCKFlute” R package [24].

### Integration of the PPI network and hub gene identification

The Search Tool for the Retrieval of Interacting Genes (STRING) [25] is a biological database for predicting protein interactions. The interactions between DEGs were evaluated using STRING, and gene sets with a combined score > 0.9 were defined as key DEGs. Subsequently, Cytoscape [26] (version 3.6.1; http://cytoscape.org/) was used to visualize the PPI network of the key DEGs that were identified. cytoHubba [27], a Cytoscape plugin, was used to extract the hub genes. The central elements were ranked by betweenness.

### Identification of putative target genes of JUN

We searched public ChIP-seq data of *JUN* on the Cistrome Data Browser (http://cistrome.org/db) [28], a website that collected and integrated thousands of public ChIP-seq data of both human and mouse. We identified and downloaded 25 processed ChIP-seq data of *JUN* on Cistrome Data Browser. Genes with high RP scores had high likelihoods to be the target genes of *JUN*. We selected genes with mean RP scores > 1 as candidate target genes of *JUN*. For further filtering of these candidate genes, we performed co-expression analysis to identify genes that were co-expressed with *JUN*. Genes with absolute correlation value > 0.5 in each dataset were selected as co-expressed genes. Candidate target genes that were co-expressed in over half of the expression datasets were defined as putative target genes of *JUN*.

### Drug repurposing analysis

Genes that were differentially expressed between OA and normal from both synovial and cartilage tissues were used for drug repurposing analysis. The probe name of the upregulated and downregulated genes were used as input on the cMap (https://portals.broadinstitute.org/cmap/) website. The “quick query” mode of the cMap algorithm was used for the drug repurposing analysis.

### Statistical analysis

Statistical analyses were performed using the R software (http://www.R-project.org/). Statistical analyses gathering more than two groups were performed using ANOVA. Otherwise, for two groups, statistical analyses were performed using the unpaired *t* test. Multiple hypothesis testing corrections were applied where multiple hypotheses were tested and were indicated by the use of FDR.

## Results

### Outline of data analysis

**Fig. 1:**
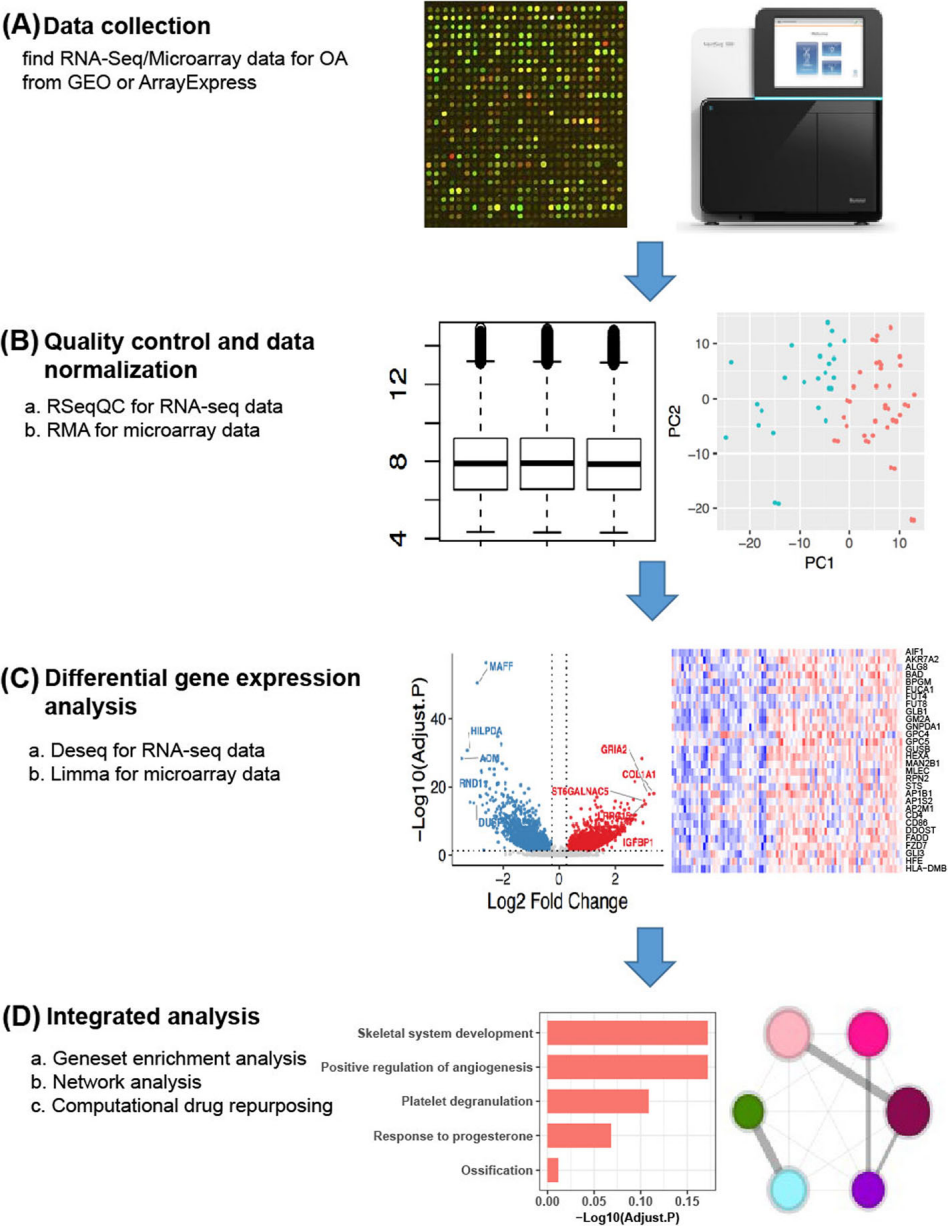
Overview of data processing step. **a** Transcriptomic data from 7 independent studies were selected, which included 132 OA and 74 normal samples. **b** Quality control and normalization for each data set. **c** Differential gene expression analysis of OA compared with normal was performed using “Limma” (for microarray data) or “Deseq2” (for RNA-seq data). **d** Strategies for integrated analysis

The objective of this study was to use differentially expressed genes (DEGs) to identify dysregulated genes and pathways of OA and to uncover potential novel pharmacological strategies. To achieve this purpose, we collected hundreds of transcriptome data of patients with OA and normal tissues from 7 independent datasets (Table 1). The subsequent analyses focused on prioritizing key regulators by protein–protein interaction (PPI) network and identifying potent drugs that can be repurposed to treat OA by the computational drug repurposing method (Fig. 1).

**Table 1:** Information about the collected datasets

	1	2	3	4	5	6	7
Data	GSE55457 [29]	GSE55235 [29]	GSE12021 [30]	GSE1919 [31]	GSE117999	GSE114007 [32]	E-MTAB-6266 [33]
Type	Array	Array	Array	Array	Array	RNA-Seq	RNA-Seq
Tissue	Synovial	Synovial	Synovial	Synovial	Cartilage	Cartilage	Cartilage
Normal	10	10	9	5	12	18	10
OA	10	10	10	5	12	20	65

### Molecular characteristic of OA

**Fig. 2:**
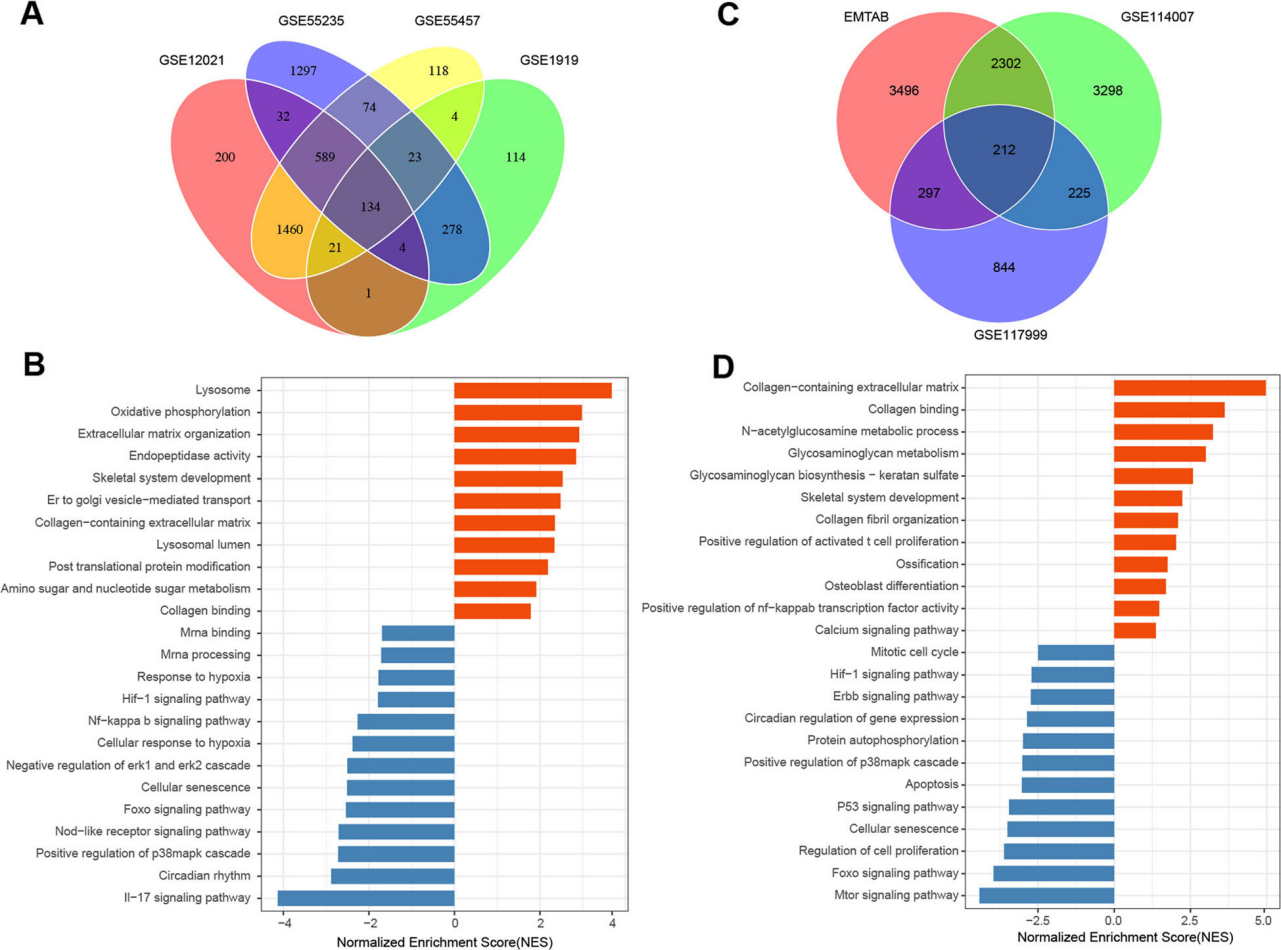
Gene ontology analysis of DEGs from synovial and cartilage tissues. **a** Venn plot of DEGs from synovial transcriptome datasets. **b** Gene ontology analysis of genes that were differentially expressed in more than two of the four synovial transcriptome datasets. “MAGeCKFlute” R package was applied to perform the enrichment analysis. **c** Venn plot of DEGs from cartilage transcriptome datasets. **d** Gene ontology analysis of genes that were differentially expressed in more than two of the three cartilage transcriptome datasets. “MAGeCKFlute” R package was applied to perform the enrichment analysis

To get a list of OA-related DEGs, we compared gene expression profiles of OA patients with those of normal tissues. We collected transcriptome datasets from synovial or cartilage tissues of OA patients and healthy donors. To distinguish DEGs between OA and normal, we performed the differential analysis of each dataset. Among the four transcriptome datasets of synovial tissues, there were 579 DEGs from GSE1919, 2441 DEGs from GSE12021, 2431 DEGs from GSE55235, and 2423 DEGs from GSE55457. The overlap of the DEGs between each dataset was shown in Fig. 2a. Genes that were differentially expressed in two of the four datasets were selected for gene set enrichment analysis. Genes associated with the lysosome, oxidative phosphorylation, extracellular matrix organization, endopeptidase activity, skeletal system development, and collagen-containing extracellular matrix were upregulated in synovial tissues of OA patients than healthy donors (Fig. 2b). In contrast, compared with normal tissues, genes related to IL-17 signaling pathway, circadian rhythm, positive regulation of p38mapk cascade, NOD-like receptor signaling pathway, Foxo signaling pathway, cellular senescence, negative regulation of erk1 and erk2 cascade pathway, and cellular response to hypoxia were downregulated in patients with OA (Fig. 2b).

To distinguish whether the cartilage tissues had different gene expression patterns from what we have observed in synovial tissues, we also collected three transcriptome datasets from cartilage tissues. By comparing the gene expression pattern between patients with OA and healthy donors, we obtained 6307 DEGs from E-MTAB-6266, 6037 DEGs from GSE114007, and 1578 DEGs from GSE117999. The overlap of the DEGs among these three datasets was shown in Fig. 2c. Genes that were differentially expressed in two of the three datasets were selected for further analysis. Gene ontology analysis of the DEGs revealed that genes involved in collagen-containing extracellular matrix, collagen binding, N-acetyglucosamine metabolic process, collagen fibril organization, skeletal system development, ossification, and osteoblast differentiation were upregulated in OA samples (Fig. 2d); while biological processes, including mTOR signaling pathway, Foxo signaling pathway, regulation of cell proliferation, cellular senescence, positive regulation of p38mapk cascade, and ERBB signaling pathway, were downregulated in OA samples (Fig. 2d). Although there were uniquely enriched pathways for synovial and cartilage tissues, we also found some pathways that were enriched in both synovial and cartilage tissues. Such pathways might play essential roles in the development and pathology of OA.

**Fig. 3:**
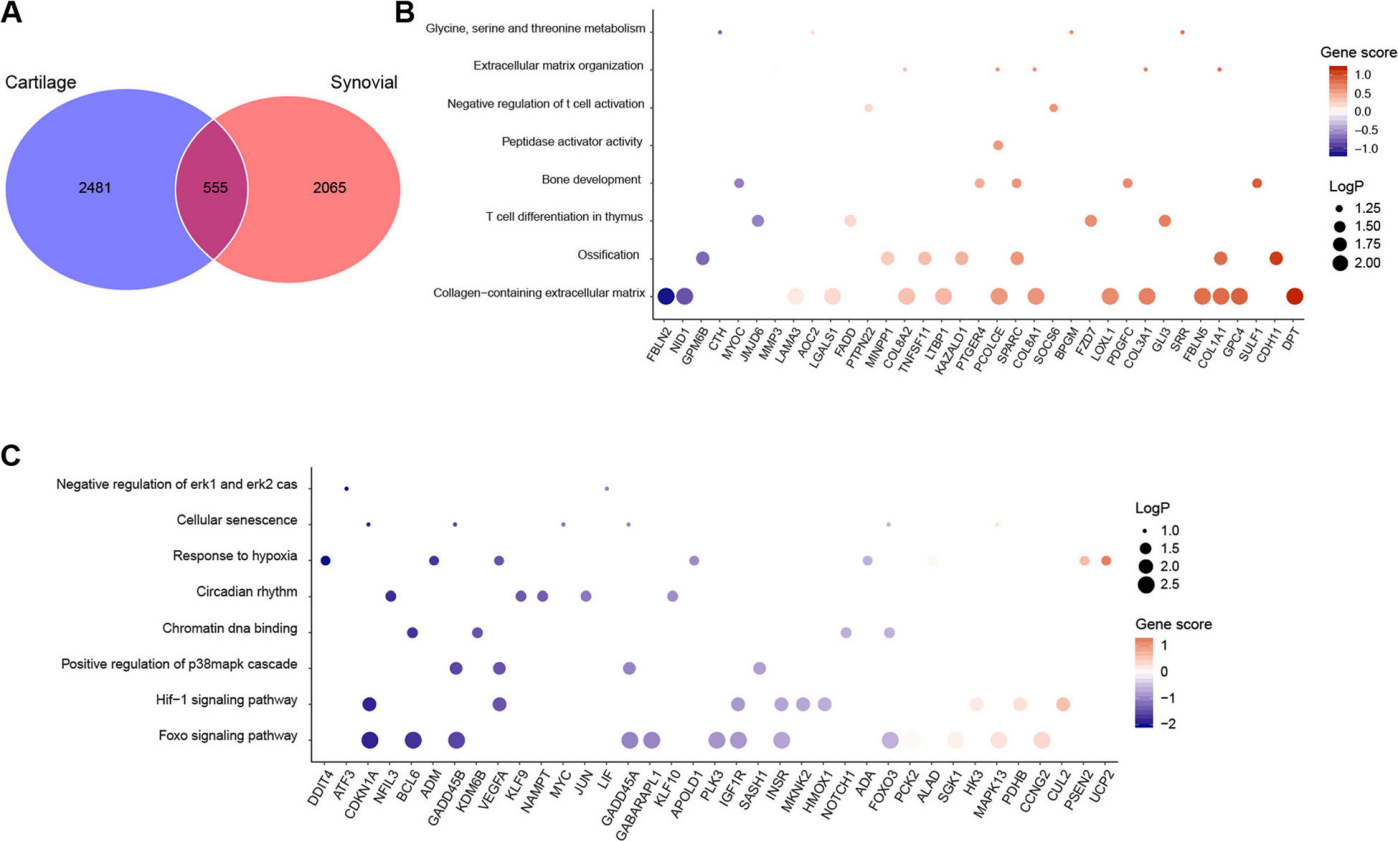
Gene ontology analysis of common DEGs from synovial and cartilage tissues. **a** Venn plot of DEGs from synovial and cartilage transcriptome datasets. **b** Gene ontology analysis of genes that were upregulated in both synovial and cartilage transcriptome datasets comparing OA with normal. “MAGeCKFlute” R package was applied to perform the enrichment analysis. **c** Gene ontology analysis of genes that were downregulated in both synovial and cartilage transcriptome datasets comparing OA with normal. “MAGeCKFlute” R package was applied to perform the enrichment analysis

To better understand the differences between OA and normal on the molecular expression pattern, we focused on the genes that were differentially expressed in both synovial and cartilage tissues for further analysis. Among the 3036 DEGs of cartilage and 2620 DEGs of synovial, there were 555 genes differentially expressed in both tissues (Fig. 3a*,* Supplementary table 1). Next, we performed functional enrichment analysis for these 555 common DEGs. Compared with normal tissues, genes involved in glycine, serine and threonine metabolism, extracellular matrix organization, regulation of T cell activation, peptidase activator activity, bone development, ossification, and collagen-containing extracellular matrix were enriched in OA samples (Fig. 3b), while pathways related to negative regulation of erk1 and erk2 cascade, cellular senescence, response to hypoxia, circadian rhythm, positive regulation of p38mapk cascade, and HIF-1 signaling pathway were downregulated in OA samples (Fig. 3c). Genes involved in these pathways might be responsible for the cartilage homeostasis and the pathology of OA.

### Protein–protein interaction network of DEGs

**Fig. 4:**
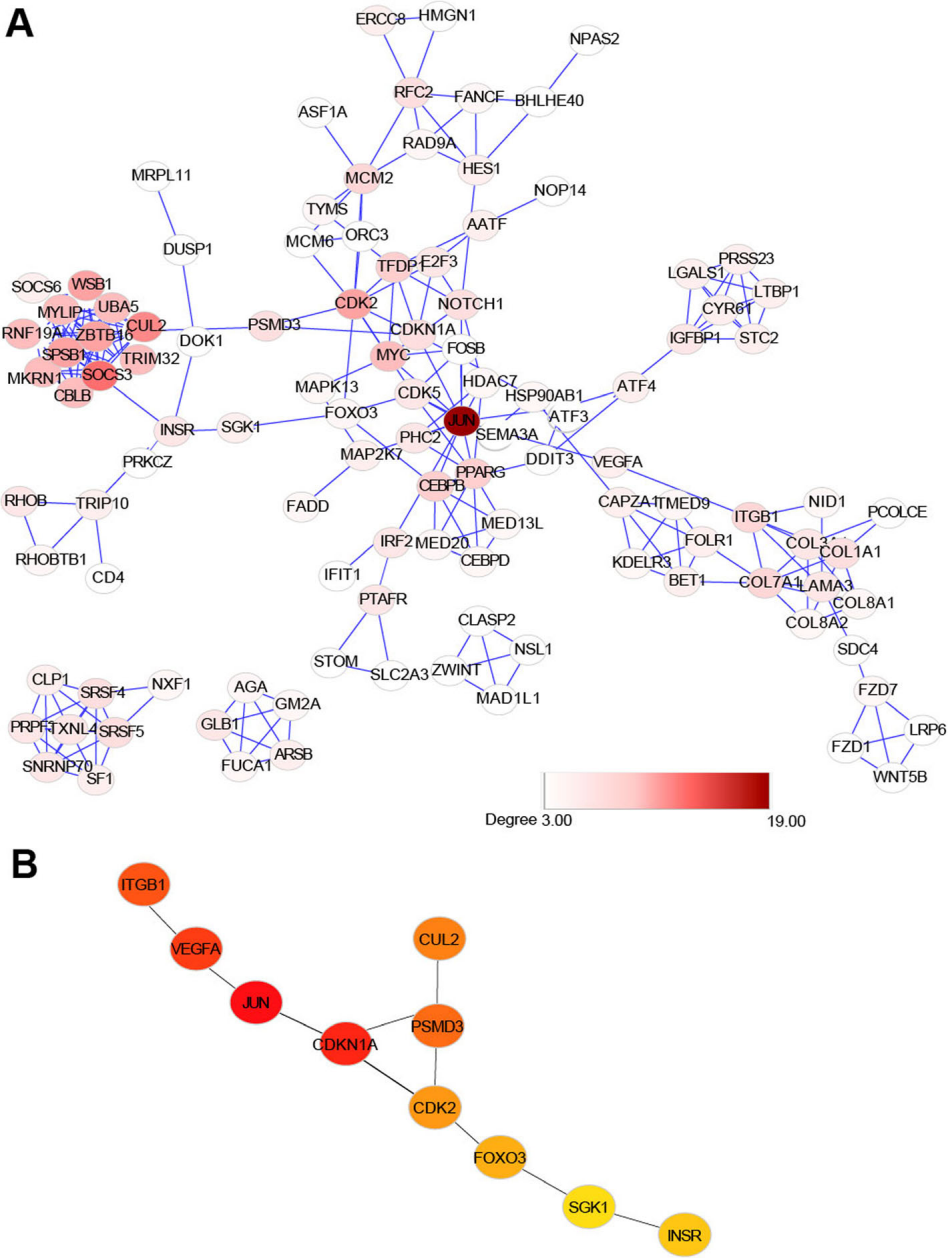
Protein–protein interaction network of DEGs. **a** STRING was used to evaluate protein interaction of DEGs between OA and normal. The interaction was visualized by Cytoscape. **b** Top 10 hub genes ranked by betweenness. cytoHubba was used to extract the hub genes in the network

To discover potential critical regulators of OA from DEGs, we performed protein–protein interaction (PPI) network analysis (Fig. 4a). We used cytoHubba, a Cytoscape plugin, to extract the hub genes in the network. The top 10 hub genes were *JUN*, *ITGB1*, *VEGFA*, *CDKN1A*, *CDK2*, *PSMD3*, *CUL2*, *FOXO3, SGK1,* and *INSR* (Fig.4b). These hub genes might associate with the development and pathogenesis of OA. *JUN* is a transcription factor that plays an essential role in regulating cellular proliferation and apoptosis. *JUN* is in the center of the network and interacts with a large number of DEGs in the network, suggesting the crucial role of *JUN* in regulating the expression of these DEGs. Integrins are major surface receptors of chondrocytes, and integrin β1 (*ITGB1*) has been proven to suppress chondrocyte hypertrophy and accelerate chondrocyte proliferation by in vitro studies and mouse models [34–37], suggesting that *ITGB1* is involved in OA progression and OA-induced cartilage degradation. *VEGF-A* is the founding member of the *VEGF* family and is classically referred to as *VEGF* [38]. As reported, the expression levels of *VEGF* were associated with OA progression and OA-specific pathologies, such as cartilage degeneration, osteophyte formation, synovitis, and pain. Moreover, a wide range of studies suggested that inhibition of *VEGF* signaling reduces OA progression and benefits patients with OA [39]. *CDKN1A* encodes a potent cyclin-dependent kinase inhibitor and functions as a regulator of cell cycle progression at G1. Shinsuke et al. reported that *CDKN1A*-deficient mice were more susceptible to OA-related changes in vivo, suggesting that *CDKN1A* modulation might constitute a possible therapeutic strategy for OA treatment [40]. *FOXO3* belongs to the forkhead family of transcription factors, which are characterized by a distinct forkhead domain [41]. *FOXO3* functions as a trigger for apoptosis by regulating the expression of genes necessary for cell death [42]. Akasaki et al. reported that normal articular cartilage had a tissue-specific signature of *FOXO1* and *FOXO3* but not *FOXO4* proteins. In OA cartilage, chondrocytes showed altered *FOXO* activation, which suggested *FOXO* might play a role in OA progression [43]. *SGK1* belongs to the serine/threonine-protein kinase subfamily, which contributes to the regulation of a wide variety of physiological activities, such as membrane transports, cell growth, proliferation, and apoptosis [44]. Knockdown of *SGK1* alleviates the IL-1β-induced chondrocyte anabolic and catabolic imbalance by activating autophagy in human chondrocytes [45]. Downregulation of SGK1 attenuates OA progression [46]. The biological role of these hub genes in the development and progression of OA had been studied previously, suggesting that targeting these hub genes might be an optimal novel treatment for OA. However, more experiments were needed to better elucidate the mechanism of action of these hub genes.

### JUN functions as a key TF that associated with the development of OA

**Fig. 5:**
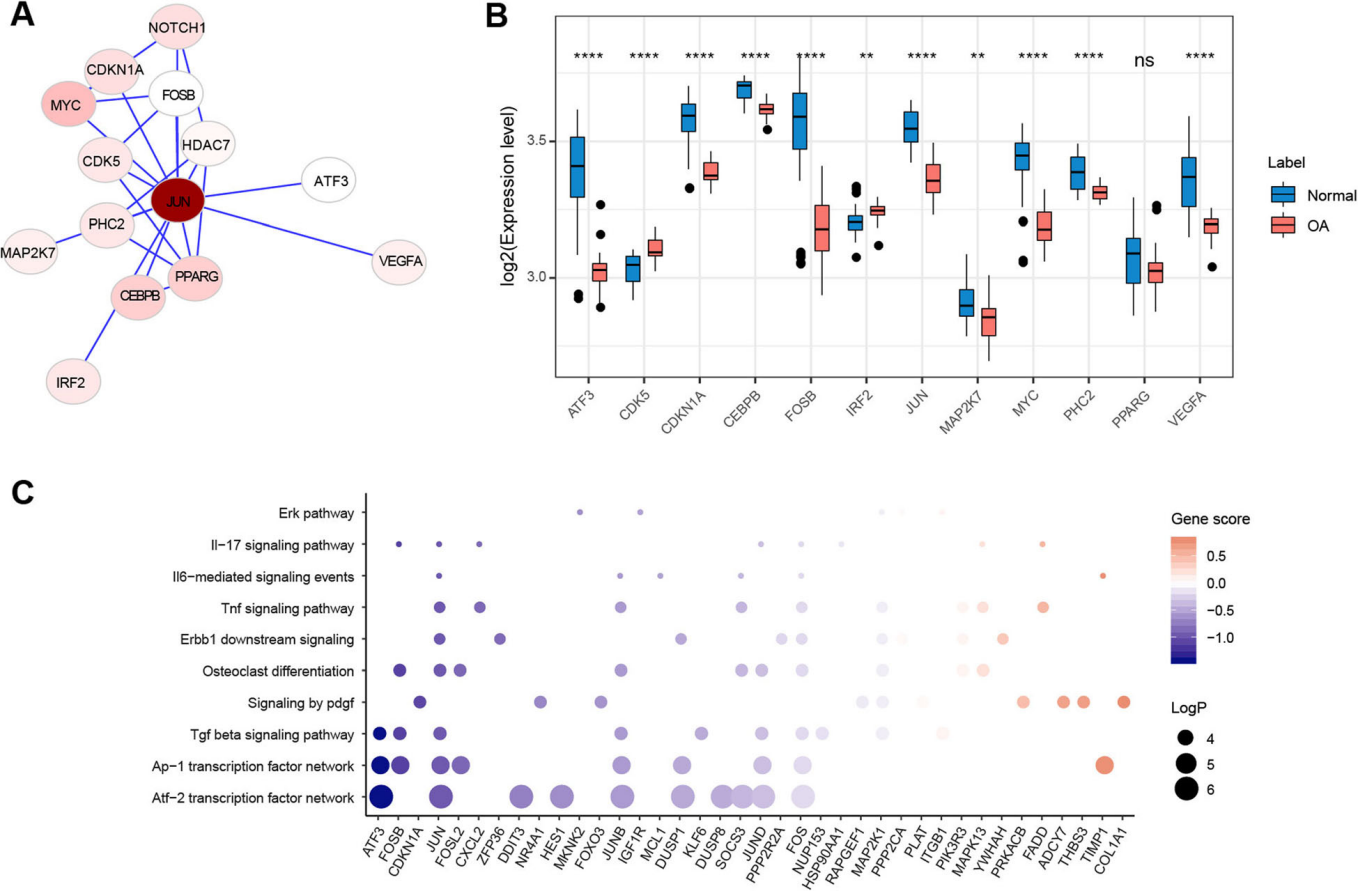
JUN functions as a key TF that associated with the development of OA. **a** A subnetwork of the first-neighbored genes with JUN. **b** The expression level of genes in the subnetwork in (**a**). Values represent mean ± s.d. *p < 0.05; **p < 0.01; ***p < 0.001; ****p < 0.0001 by Student’s *t* test. **c** Gene ontology for putative target genes of JUN. “MAGeCKFlute” R package was applied to perform the enrichment analysis

Since *JUN* was in the central of the network (Fig. 4a), we focused on *JUN* for further analysis. We found that most genes interacting with *JUN* directly in the PPI network were differentially expressed between OA and normal (Fig. 5a and b), suggesting the importance of *JUN* in regulating the expression of these DEGs. To identify the potential target genes that were regulated by *JUN*, we downloaded the processed ChIP-seq data of *JUN* on the Cistrome Data Browser (http://cistrome.org/db) [28]. Cistrome Data Browser collected thousands of human and mouse samples with ChIP-seq data and facilitated searches for putative target genes of transcription factors by regulatory potential model [47]. Regulatory potential (RP) score for each gene was calculated, reflecting the likelihood of the transcription factor being a direct regulator of that gene, and genes with high RP scores were putative targets of the transcription factor. By analyzing 25 public ChIP-seq data of *JUN*, we selected 3250 genes (Supplementary table 2) with mean RP scores > 1 as candidate target genes of *JUN*. For further filtering of the candidate genes, we performed co-expression analysis. Among the 3250 candidate targets, 214 genes had high correlations with *JUN*, which were selected for further analysis (Supplementary table 3). Functional enrichment analysis of the selected 214 genes revealed that pathways including the ATF-2 transcription factor network (part of the AP-1 complex), AP-1 transcription factor network, TGF-β signaling pathway, osteoclast differentiation, and ERBB1 downstream signaling were enriched (Fig. 5c). JUN was in the AP-1 protein family, and the top 2 enriched pathways were associated with the AP-1 family network, which suggested the creditability of our data analysis method. Among these, the TGF-β signaling pathway has been reported to play a critical role in the development and progression of OA by driving chondrocytes toward hypertrophy, promoting osteoprogenitor cell differentiation into osteoblasts, mediating synthesis of cartilage-specific extracellular matrix components, and angiogenesis in subchondral bone [48]. The effects of *TGF-β* on the modulation of extracellular matrix components were dependent on the activation of JNK (c-Jun N-terminal Kinase), which in turn modulates the activity of c-Jun [49]. Thus, *JUN* might serve as a critical regulator of the development of OA by regulating the activity of TGF-β.

### Drug repurposing for OA

Since there are no effective interventions to decelerate the progression of OA now, drug repurposing of the Food and Drug Administration (FDA)-approved therapeutic agents is a particularly attractive approach to improve the treatment of OA. Computational techniques for predictive repurposing offer a relatively efficient and authentic method of identifying testable hypotheses that may be translated into the clinic [50]. The Connectivity Map (cMap), which was established by the Broad Institute, consists of gene expression data generated by dosing of more than 1300 compounds in hundreds of cell lines [51]. cMap (https://portals.broadinstitute.org/cmap/) has been successfully used to make drug repurposing predictions for a number of disease conditions [50]. We applied cMap to explore potential drug repositioning opportunities for OA. Among the top 15 listed drugs, MG-262 ranked first (Table 2). MG-262 is a potent proteasome inhibitor that selectively and reversibly inhibits the chymotryptic activity of the proteasome [52, 53]. The proteasome inhibitors have shown anti-inflammatory activities in the animal models of arthritis, psoriasis, colitis, and other inflammatory conditions [54]. Some studies discovered that proteasome inhibitors promoted bone growth in a cell-based screen [55]. Although the biological function of MG-262 in the treatment of OA still remains an unexploited field, this analysis provided new possibilities for the treatment of OA. Of note, among the top 15 drugs, there were four drugs classified as cardiac glycosides, such as ouabain and digoxin, suggesting the potential clinical implications of cardiac glycosides in OA. Cardiac glycosides function by inhibiting the Na^+^/K^+^ ATPase (NKA) [56]. In addition, cardiac glycosides have been found to decrease inflammatory symptoms in different animal models of acute and chronic inflammation [57]. Thus, cardiac glycosides might have therapeutic benefit in the treatment of OA by countering the inflammation-induced articular cartilage degradation. Since the safety of FDA-approved drugs has been sufficiently verified, drug repurposing for approved drugs offers a less risky and more rapid potential for the investment of novel therapeutic strategies.

**Table 2:** Top 15 drugs predicted by cMap

Rank	cMap name	Enrichment	*p*	Specificity	Percent non-null	Description
1	MG-262	− 0.992	0	0	100	Inhibitor of the chymotryptic activity of the proteasome
2	Anisomycin	− 0.981	0	0.0085	100	Antibiotic, inhibiting eukaryotic protein synthesis
3	**Digoxin**	**− 0.978**	**0**	**0**	**100**	**Cardiac glycoside, inhibiting the Na**^**+**^**/K**^**+**^ **ATPase**
4	**Ouabain**	**− 0.977**	**0**	**0.0088**	**100**	**Cardiac glycoside, inhibiting the Na**^**+**^**/K**^**+**^ **ATPase**
5	Cephaeline	− 0.955	0	0.0121	100	Inducing vomiting by stimulating the stomach lining
6	Emetine	− 0.953	0	0.0118	100	Inducing vomiting by stimulating the stomach lining
7	Mebendazole	− 0.943	0	0	100	Broad-spectrum antihelminthic
8	Phenoxybenzamine	− 0.941	0	0.0091	100	Alpha-adrenoceptor antagonist, used as an anti-hypertensive
9	**Digitoxigenin**	**− 0.937**	**0**	**0**	**100**	**Cardiac glycoside, inhibiting the Na+/K+ ATPase**
10	Thioridazine	− 0.701	0	0.043	80	A first generation antipsychotic drug
11	15-Delta prostaglandin J2	− 0.634	0	0.0301	86	Anti-inflammatory lipid mediator
12	LY-294002	− 0.323	0.00002	0.2945	54	PI3K-AKT inhibitor
13	Lomustine	− 0.921	0.00006	0	100	An alkylating nitrosourea compound used in chemotherapy
14	**Digoxigenin**	**− 0.879**	**0.00008**	**0**	**100**	**Derivative of the cardiac glycoside digoxin**
15	Thapsigargin	− 0.964	0.00012	0.0258	100	An inhibitor of sarco endoplasmic reticulum Ca^2+^ ATPase (SERCA)

*Enrichment*: Positive enrichment scores represent that the biological state induced by the signature are sought. Likewise, if reversal or repression of the biological state encoded in the query signature is required, the enrichment scores were negative.

*p*: The Kolmogorov-Smirnov statistic is used for the significance analysis.

*Specificity*: Specificity measures the uniqueness of the connection between a perturbagen and the signature of interest. High values mean that many signatures show good connectivity with these instances. This may indicate that the connectivity is unexceptional.

*The non-null percentage*: The non-null percentage is defined as the percentage of all instances in a set of instances that share the majority non-null category of connectivity score. For example, if a perturbagen is represented by five instances, and three of those instances have a positive connectivity score, one instance has a null connectivity score and one instance has a negative connectivity score, the non-null percentage for that perturbagen in that result is 60%.

## Discussion

Here, we used a large-scale data integration method to characterize the molecular signatures of OA, which extended our understanding of the disease mechanisms. We also identified several essential regulators of OA, such as *JUN*, *VEGFA*, and *FOXO3*, which might provide a scientific rationale for the development of novel pharmacological therapies. Of note, we found *JUN*, a crucial dysregulated transcription factor, plays a central role in regulating the aberrant gene expression pattern in OA. Moreover, we used a computational drug repurposing method to identify potential FDA-approved drugs that can be repurposed to improve the treatment of OA.

In this study, we integrated and analyzed multiple published datasets. We were able to find genes that were consistently differentially expressed between OA and normal among different datasets and different tissues. Our analysis confirmed some findings from previous studies using genome-wide gene expression analyses. Common findings of these studies are the differential expression of genes involved in matrix-degrading enzymes (MMPs, ADAMTS), collagen organization, and inflammation [7, 58]. In our analysis, we also found increased expression of genes associated with collagen-containing extracellular matrix (*DPT*, *GPC4*, *COL8A2*, *FBLN5*, *COL3A1*), ossification (*CDH11*, *COL1A1*, *SPARC*, *KAZALD1*), and bone development (*DYM*, *PTGER4*, *SPARC*, *SULF*) in patients with OA, which is suggestive of active remodeling of cartilage homeostasis during OA pathogenesis. During the early stages of OA, the molecular composition and organization of the extracellular matrix are altered first [59]. The articular chondrocytes exhibit increased cell proliferation and matrix synthesis for the purpose of initiating repairing for pathological injury [59, 60]. Changes in the composition and structure of the articular cartilage further stimulate chondrocytes to produce more catabolic factors involved in cartilage degradation. Thus, the expression of genes involved in the carbohydrate metabolism and extracellular matrix components were upregulated. Moreover, chronic low-grade inflammation has also been found to contribute to the development and progression of OA [61]. During OA progression, the entire synovial joints were involved in the inflammation process [62]. Pro-inflammatory factors, such as IL-1β and TNF-α, as well as chemokines, were reported to contribute to the systemic inflammation that led to the activation of NF-κB signaling in both synovial cells and chondrocytes [63]. Based on these studies, multiple novel pharmacological strategies have emerged, including anti-inflammatory mediators (anti-IL-1 [64], anti-TNF-α [65], and anti-IL-6 [66]) and inhibition of catabolic pathways (Wnt, ADAMTS, and cathepsin K) [67]. Apart from these findings, we were able to find some essential regulators that might associate with the pathogenesis of OA, such as *ITGB1*, *PSMD3*, *CUL2,* and *INSR*. Since the association of these hub genes with the development of OA has been reported, they could serve as important diagnostic and/or therapeutic targets for OA. However, more mechanistic interpretation and clinical trial data are needed to clarify the efficacy in the treatment of OA.

Since synovial and cartilage play different roles in the bone homeostasis and pathology of OA, we compared the gene expression pattern between OA and normal using transcriptome datasets of each tissue separately. This analysis identified some tissue-specific DEGs between OA and normal. Gene sets associated with N-acetylglucosamine metabolic process, glycosaminoglycan biosynthesis and metabolism, NF-KB transcription factor activity, ERBB signaling pathway, P53 signaling pathway, and mTOR signaling pathway were uniquely differentially expressed in cartilage tissues. While biological pathways involved in lysosome, oxidative phosphorylation, endopeptidase activity, Nod-like receptor signaling pathway, and IL-17 signaling pathway were uniquely differentially expressed in synovial tissues. In the development of OA, the pathological changes of synovial and cartilage were different. The histological pattern of synovium in OA patients is characterized by synovial lining hyperplasia, increased vascularity, and sublining fibrosis [68] with the phenotypic shift of chondrocytes, including surface fibrillation, degradation of cartilage matrix, chondrocyte clusters appearance, and vascular penetration from the subchondral bone [69]. The different phenotypic changes of synovial and cartilage might explain the different OA-related DEGs between these two tissues.

Our study also identified JUN, a transcription factor, as a key regulator of these DEGs. JUN is one of the members of the Activator protein 1 (AP-1) family proteins [70]. AP-1 family proteins are basic leucine zipper (bZIP) transcription factors that consist of Jun (c-Jun, JunB, and JunD), Fos (c-Fos, FosB, Fra-1, and Fra-2), Jun dimerization partners (JDP1 and JDP2), and the closely related activating transcription factors (ATF2, LRF1/ ATF3, and B-ATF) subfamilies [71]. AP-1 family proteins are implicated in the regulation of a variety of cellular processes, including proliferation and survival, differentiation, apoptosis, cell migration, and transformation [72]. JUN has been reported to play a crucial role in regulating cell proliferation and apoptosis [73]. Ventura *et al.* reported that the c-Jun NH2-terminal kinase JNK signaling pathway contributes to the regulation of TGF-β-mediated biological responses [74]. TGF-β is crucial for cartilage maintenance, and the lack of TGF-β results in OA-like changes [74]. Thus, JUN might regulate the development of OA by coordinating with TGF-β signaling.

Drug discovery is a time-consuming, laborious, costly, and high-risk process. Drug repositioning is efficient, economical, and low-risk compared with the traditional drug development process. There are multiple drug repurposing methods generated, including computational approaches, biological experimental approaches, and mixed approaches. Among these, computational drug repurposing is the most powerful and the most widely used. Previously, Soul et al. constructed a data portal that provides an exploration and comparison platform for analyzed skeletal transcriptomics data [75]. This data portal also provides data repurposing mode using L1000 data, one of the cMap project resources. Using a computational drug repurposing method, we found cardiac glycosides might be repurposed in the treatment of OA. Cardiac glycosides are drugs that inhibit the Na^+^/K^+^ ATPases and are applied to treat heart failure and certain irregular heartbeats. However, recent studies reported that cardiac glycosides are a novel class of broad-spectrum senolytics for therapeutic applications in many age-related disorders [76], including osteoarthritis. Cardiac glycosides were capable of reducing the number of senescent cells, diminishing the level of local inflammation, and improved some metabolic and physical fitness parameters that decline with aging in some animal models [77, 78]. Drug repurposing can be highly attractive as a potentially cheaper and faster route to market. However, successful drug repositioning requires a deep understanding of biological mechanisms—from known overlaps of mechanisms to re-innovation of a new molecule, to find a new mechanism, dosing, route of administration, and new target. Although our drug repurposing analysis offers novel and valuable options for developing strategies to treat OA, the effectiveness of the predicted drugs in treating OA, such as cardiac glycosides, needs more systemic and detailed validation.

There are several limitations of our studies. First, osteoarthritis is typically described as a heterogeneous disease with complex pathogenesis. Different patients might have different mechanistic pathways, such that the mechanisms of OA in the elderly might be different from those after a joint injury in a younger adult or in obese individuals. The molecular signatures we discovered in this study by transcriptome data analysis might not be representative in some subtypes of OA patients. Integrated analysis of multi-omics data, including epigenetics (DNA methylation, histone post-translational modification, and/or non-coding RNA), metabolomics, and proteomics (by LC-MS) data, are needed to better elucidate the heterogeneity of OA. Such detailed and systematic analyses offer important mechanistic and potentially therapeutic insights into OA. Finally, we have highlighted several essential regulators and uncovered that cardiac glycosides might benefit the patients with OA by countering the inflammation. However, further experimental studies are needed to validate their clinical implications.

Our study integrated multiple public transcriptome data sets together, which provides more comprehensive and reliable insights into the genetic alterations associated with the disease phenotype. Moreover, we used a computational drug repurposing method to identify potent drug candidates to improve the treatment of OA.

## Conclusion

In summary, we used bioinformatics analysis to identify a group of differentially expressed genes between OA and normal tissues. By protein–protein interaction network analysis, we identified several osteoarthritis-related hub genes, which might be potential diagnostic or therapeutic targets for osteoarthritis. In addition, we found *JUN*, a transcription factor, functions as a crucial regulator by the analysis of public ChIP-seq data and co-expression analysis. Moreover, we used a computational drug repurposing method to identify potent drugs that can be repurposed to treat OA.

## Supplementary Information

**Additional file 1: Sup.table 1**: Differentially expressed genes (DEGs) between OA and normal.

**Additional file 2: Sup.table 2**: RP scores of public ChIP-seq data of JUN.

**Additional file 3: Sup.table 3**: Putative targets of JUN.

## Data Availability

The datasets used and/or analyzed in this study are available from the corresponding author on reasonable request.

## Abbreviations

OA: Osteoarthritis
DEGs: Differentially expressed genes
PPI: Protein–protein interaction
RMA: Robust Multi-array Average
ECM: Extracellular matrix
GO: Gene Ontology
STRING: Search Tool for the Retrieval of Interacting Genes
RP: Regulatory potential
